# Supplementary figures and images for: Multiple long-range cis interactions generate CTCF insulator-dependent viral chromatin domains in quiescent HSV-1 genomes
Source: mBio. 2025 Aug 28;16(10):e01638-25. doi: 10.1128/mbio.01638-25 (PMC12506143; doi:10.1128/mbio.01638-25)

S. Fig. 1.

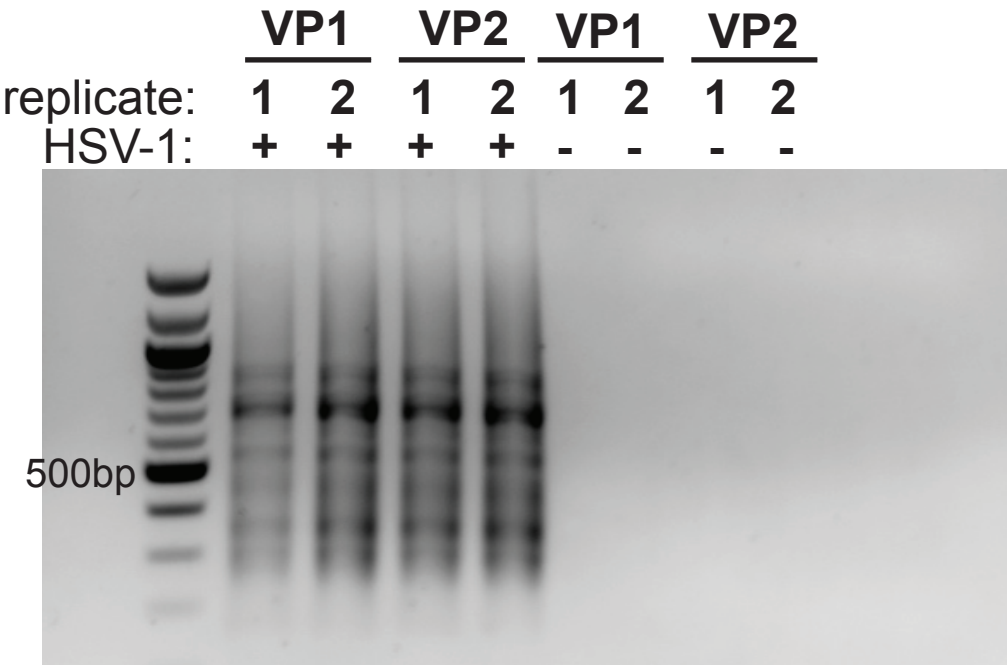

Supplement: Figure S1 — Validation of HSV-1-specific viewpoints for 4C-seq. [file mbio.01638-25-s0001.pdf]

S. Fig. 2A

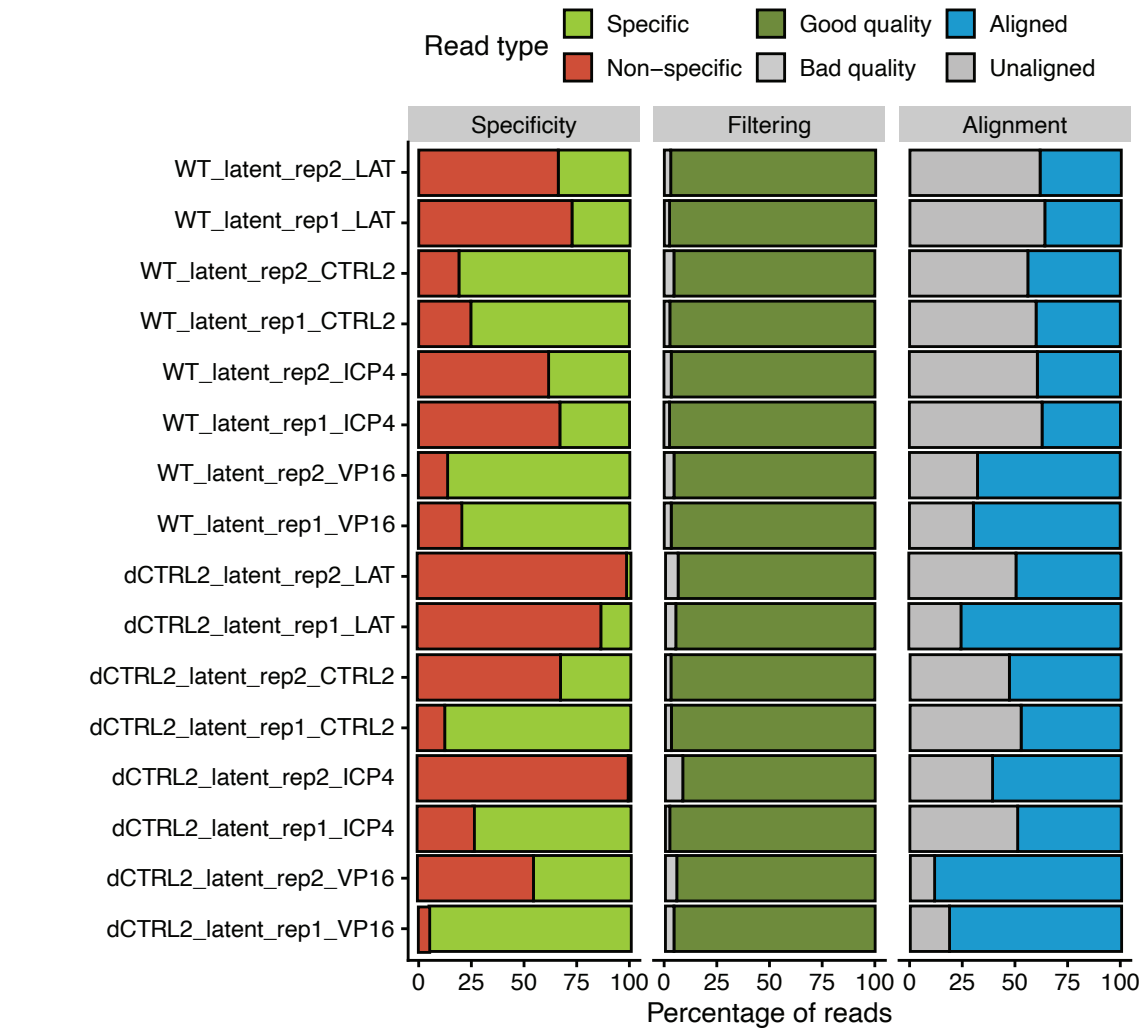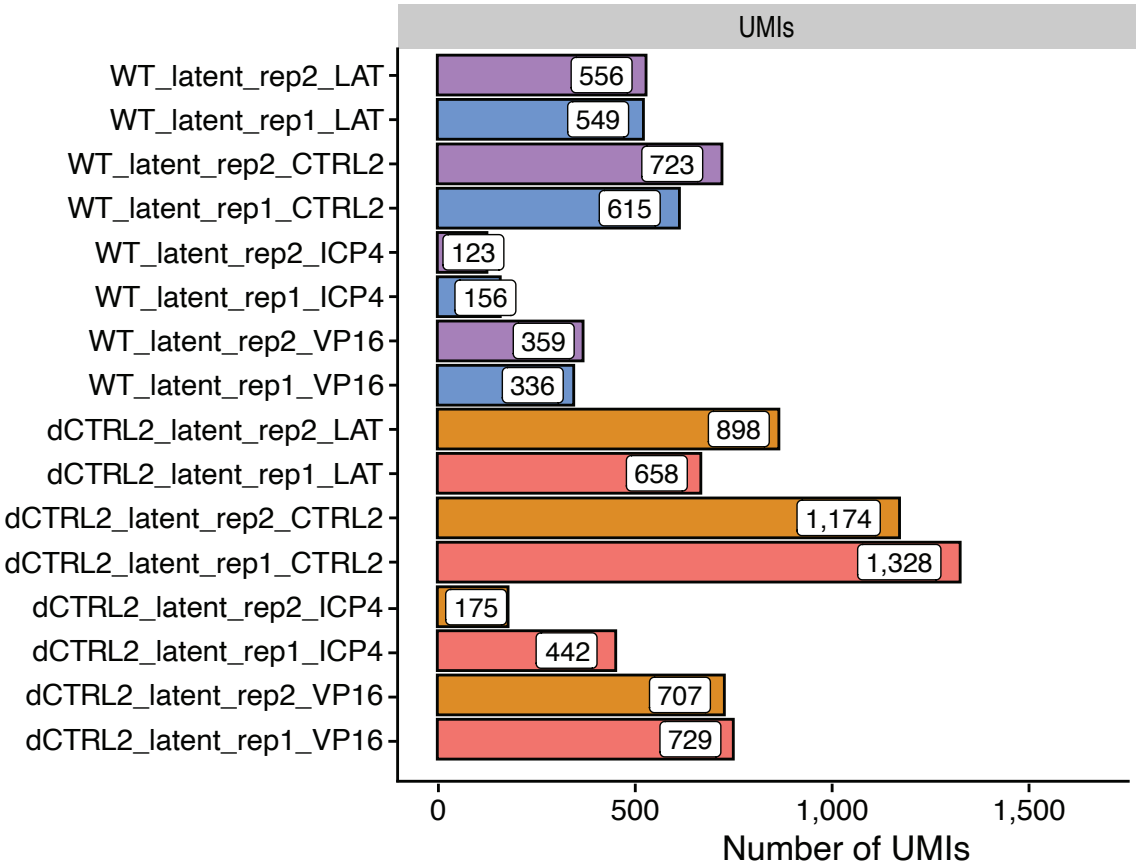

Supplement: Figure S2A — Paired-end sequencing of multiplexed UMI-4C amplicons. [file mbio.01638-25-s0002.pdf]

S. Fig. 2B

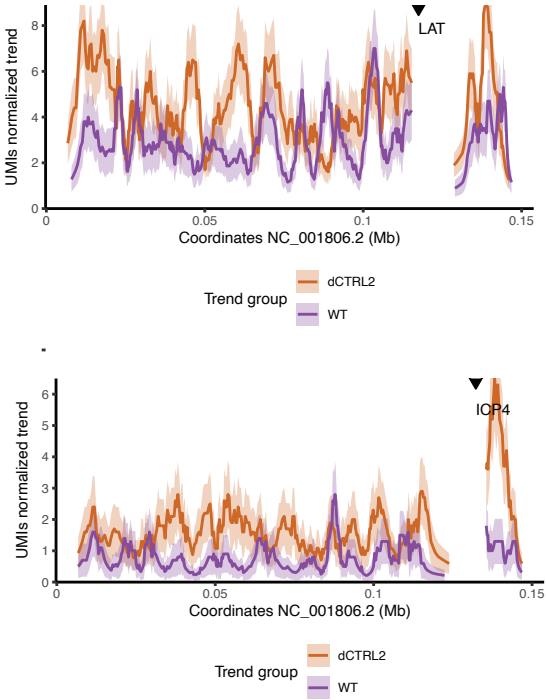

Supplement: Figure S2B — UMI-normalized interaction frequency profiles. [file mbio.01638-25-s0003.pdf]

S. Fig. 2C

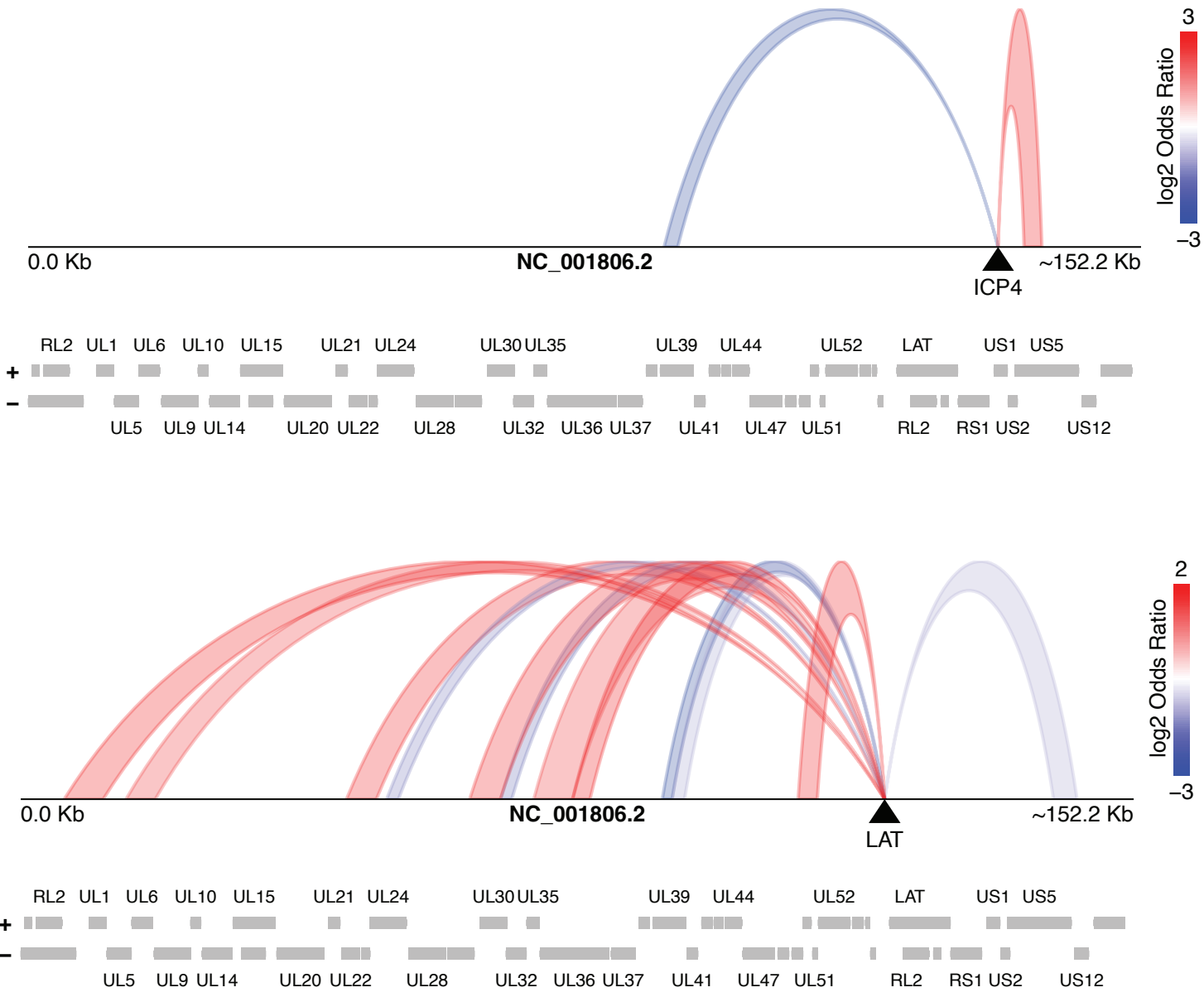

Supplement: Figure S2C — Differential chromatin interaction arcs. [file mbio.01638-25-s0004.pdf]
