# Supplementary material for: Multiple long-range cis interactions generate CTCF insulator-dependent viral chromatin domains in quiescent HSV-1 genomes
Source: mBio. 2025 Aug 28;16(10):e01638-25. doi: 10.1128/mbio.01638-25 (PMC12506143; doi:10.1128/mbio.01638-25)
Supplement: Legends — Supplemental figure legends. [file mbio.01638-25-s0005.docx]

**Supplemental Figures**

**S. Figure 1.** Validation of HSV-1-specific viewpoints for 4C-*seq*. PCR amplification of the bait-targeted viewpoints 1 (VP1) and 2 (VP2) from HSV-1-infected (+) and uninfected (-) 4C templates was performed across three replicates (only first two are shown in the figure). PCR products were resolved on a 1.5% agarose gel, with a DNA ladder shown as a size reference. Amplification was successful in HSV-1-infected samples for both VP1 and VP2, while no amplification was observed in uninfected samples, confirming the specificity of the viewpoints to HSV-1-infected templates.

**S. Figure 2A:** Paired-end sequencing of multiplexed UMI-4C amplicons resulted in a similar number of reads with a relatively balanced distribution of UMIs and a high degree of consistency across replicates across each bait.

**S. Figure 2B:** UMI-normalized interaction frequency profiles across the HSV-1 genome for LAT viewpoints (top panel) and ICP4 (bottom panel) in wt and ΔCTRL2-infected LUHMES cells. Increased interaction frequencies and broader interaction domains are observed in ΔCTRL2 genomes compared to wt.

**S. Figure 2C:** Differential chromatin interaction arcs for ICP4 (top panel) and LAT (bottom panel) viewpoints visualized as arc plots across the HSV-1 genome. Arcs represent statistically significant changes in *cis*-interactions peaks between ΔCTRL2 and wt genomes. Red arcs indicate **increased** interaction frequencies in ΔCTRL2 relative to wt (log₂ odds ratio > 1), while blue arcs indicate **reduced** interactions. HSV-1 genome coordinates and annotated ORFs are displayed below the arc plots.
